# Disturbances and Associated Factors Among Children With Autism Spectrum Disorder: A Cross-Sectional Study

**DOI:** 10.7759/cureus.101920

**Published:** 2026-01-20

**Authors:** Hussain A Al Ghadeer, Hussain A Al Ibrahim, Ahmed S AlKhars, Fatimah H Al Hamad, Ali I Al Ali, Zinab A Al Bosrour, Doaa Z Fadaaq, Norah S Alibrahim, Sarah A Almousa, Faisal H Alqahtani, Khawla A Al Hassan

**Affiliations:** 1 Paediatrics, Maternity and Children Hospital, AlAhsa, SAU; 2 Psychiatry, Maternity and Children Hospital, AlAhsa, SAU; 3 Paediatrics, European Medical Center, Jeddah, SAU; 4 Paediatrics, King Faisal University, AlAhsa, SAU

**Keywords:** alahsa., autism spectrum disorder (asd), saudi arabia, sleep disorder, sleep hygiene

## Abstract

Introduction

Children with autism spectrum disorder (ASD) experience a range of comorbidities, including sleep disorders. Sleep disturbances are more prevalent in this population than in typically developing children and may exacerbate behavioral symptoms such as inattention and irritability, increasing the overall psychosocial burden of ASD.

Aim

This study assessed the pattern, frequency, and predictors of sleep disturbances among children with ASD in Al-Ahsa, Saudi Arabia.

Methods

A cross-sectional study was conducted between April 2025 and October 2025 among children diagnosed with ASD at the Child Development Center of Maternity and Children Hospital, Al-Ahsa. Caregivers completed the Children’s Sleep Habits Questionnaire (CSHQ), a validated 33-item instrument covering eight sleep domains, with a total score ≥41 indicating clinically significant sleep disturbance. Additional demographic, behavioral, and clinical data were collected. Descriptive statistics, *t*-tests, one-way ANOVA, and multiple linear regression were used to identify factors associated with sleep disturbance.

Results

A total of 116 children with ASD were included, with a mean age of 6.2±1.9 years. The mean total CSHQ score was 76.3±9.3, indicating a high burden of sleep disturbances. In bivariate analyses, total CSHQ scores were significantly associated with age at autism diagnosis, autism severity, parental sleep-related anxiety, sleeping arrangement, caffeine intake, and history of sleep medication use. In multivariable linear regression analysis, age at autism diagnosis, autism severity, parental sleep-related anxiety, sleeping arrangement, and prior use of sleep-inducing medications remained independently associated with total sleep disturbance scores, whereas caffeine intake was not retained in the final model.

Conclusion

Sleep disturbances were highly prevalent among children with ASD, affecting multiple sleep domains and influenced by a combination of clinical and psychosocial factors. Routine sleep screening, caregiver education, early ASD diagnosis, and integrated behavioral and medical interventions are essential to improving sleep and overall quality of life in this population.

## Introduction

Deficits in communication, social interaction, and restricted, repetitive patterns of behavior are considered the core hallmarks of autism spectrum disorder (ASD) [1]. Globally, ASD is diagnosed in approximately one in 100 children. Prevalence estimates vary widely both within and between sociodemographic groups and have increased over time, partly due to changes in diagnostic criteria, increased awareness, and improved case ascertainment [2]. This rise may be attributed to several factors, including expansion of the diagnostic criteria, greater public awareness, and improved recognition of ASD by healthcare practitioners [3]. Consequently, determining the true prevalence of ASD remains challenging. Chiarotti and Venerosi [3] highlighted this variability in a recent global narrative review, demonstrating substantial differences in prevalence across countries. Reported estimates range from 0.42% to 3.13% in Europe, 0.11% to 1.53% in the Middle East, 0.08% to 9.3% in Asia, and 0.87% to 1.85% in North America. In Saudi Arabia, ASD is estimated to affect 2.81 per 1000 children [4].

Co-morbid mental health conditions are frequently observed in individuals with ASD. A large population-based study reported that up to 94% of individuals with ASD have at least one comorbid psychiatric diagnosis, with an overall comorbidity prevalence of approximately 54.8%. Commonly reported conditions include attention-deficit/hyperactivity disorder (ADHD), anxiety disorders, depressive disorders, and sleep disturbances [5,6]. In addition, systemic comorbidities such as epilepsy, gastrointestinal (GI) disorders, and hearing or vision impairments occur more frequently among children with ASD. Early identification and management of these conditions facilitate improved outcomes and more effective interventions [7,8].

Sleep disorders represent one of the most common and impactful comorbidities associated with ASD. Their prevalence in children with ASD ranges from 45% to 86% [8,9]. Compared with typically developing children, who show sleep difficulties in 11% to 37% of cases, children with ASD are 50% to 80% more likely to experience sleep problems [10]. These disturbances are thought to result from factors such as sensory hyperarousal, circadian rhythm abnormalities, alterations in melatonin pathways, psychiatric comorbidities, and environmental influences [10]. Sleep difficulties also vary across developmental stages: children often exhibit bedtime resistance, nighttime awakenings, and parasomnias, while adolescents tend to show delayed sleep onset, reduced total sleep time, and increased daytime sleepiness [11,12].

Adequate sleep is essential for optimal physical growth, emotional regulation, and cognitive functioning in children. Sleep disturbances encompass a broad range of problems, including difficulties initiating sleep (sleep-onset delay), resistance to bedtime routines, insufficient sleep duration, night waking, parasomnias, sleep-disordered breathing, and excessive daytime sleepiness. These disturbances are particularly common in children with ASD and correspond to the domains assessed by the Children’s Sleep Habits Questionnaire (CSHQ). Emerging evidence indicates that sleep disturbances in children with ASD are associated with worsening core autism symptoms, impaired attention and executive functioning, reduced academic performance, and increased behavioral and emotional difficulties, including irritability, hyperactivity, and anxiety [13,14]. Additionally, sleep problems in children with ASD are associated with elevated parental stress and depressive symptoms, which may further disrupt overall family functioning. Given their broad impact on both child and family well-being, sleep disturbances in ASD require careful clinical attention. Behavioral interventions and, when necessary, pharmacological treatments are commonly used to manage sleep problems in this population [15].

Despite the high prevalence and clinical significance of sleep disturbances in children with ASD, data from Middle Eastern and Saudi populations remain limited, and few studies have comprehensively examined the persistence and severity of sleep problems alongside modifiable clinical, demographic, and behavioral factors. Understanding the chronicity of sleep disturbances in children with ASD is essential for identifying targets for early intervention that may improve sleep quality, ASD-related symptoms, and overall quality of life. Therefore, this study aimed to examine the patterns and severity of sleep disturbances among children with ASD in Al-Ahsa, Saudi Arabia, and to identify the clinical, demographic, and behavioral factors associated with increased sleep difficulties.

## Materials and methods

Study design and population

This cross-sectional, observational study was conducted between April 2025 and October 2025 at the Child Development Center of Maternity and Children Hospital, Al-Ahsa, Saudi Arabia. The study participants were parents or primary caregivers of children with ASD, who provided information regarding their children’s sleep behaviors and related factors. Eligible children were aged 2-14 years and had a confirmed clinical diagnosis of ASD established by qualified treating physicians at the Child Development Center based on standardized clinical assessment consistent with internationally accepted diagnostic guidelines and routine institutional practice. The diagnosis was made as part of routine clinical care and was neither performed nor reassessed by the research team, and no additional psychometric testing was conducted for research purposes.

Children of both sexes who had been regularly followed at the center for at least one year were included. Exclusion criteria comprised children younger than two years or older than 14 years, those with known organic neurological disorders, significant psychiatric comorbidities, intellectual disability, chromosomal abnormalities, or irregular follow-up at the center. Eligible respondents were parents or primary caregivers who lived with the child, were directly involved in daily care, and were able to reliably report the child’s sleep behaviors and daily routines. Caregivers who were not the primary source of daily care or who were unable to complete the questionnaire were excluded.

The study protocol was reviewed and approved by the Institutional Review Board of Maternity and Children Hospital, Al-Ahsa, Saudi Arabia (IRB approval No. H-05-HS-137). Written informed consent was obtained from all participating caregivers before enrollment.

Data collection

This study was purely observational and non-interventional. The researchers did not modify patient care, influence treatment decisions, or introduce any experimental procedures. Data were collected exclusively through caregiver-reported questionnaires reflecting routine clinical practice. Caregivers completed a structured questionnaire that included the CSHQ in addition to investigator-designed items collecting demographic, lifestyle, and clinical information. Before questionnaire completion, caregivers received brief standardized instructions from the research team explaining the purpose of the study and how to complete the questionnaire based on their child’s usual sleep patterns over recent weeks. No direct observation of sleep behaviors was performed by the research team, and data were not obtained through medical chart review.

Parental anxiety regarding the child’s sleep was assessed using a single caregiver-reported item included in the investigator-designed questionnaire. Caregivers were asked whether they felt anxious or worried about their child’s sleep problems (yes/no). This item was intended to capture perceived caregiver concern rather than to diagnose anxiety using a validated psychological scale.

CSHQ

Sleep behaviors were assessed using the CSHQ [16], a widely used and validated parent-reported instrument designed to screen for sleep disturbances in children. The questionnaire consists of 33 items distributed across eight domains: bedtime resistance (6 items), sleep-onset delay (1 item), sleep duration (3 items), sleep anxiety (4 items), night waking (3 items), parasomnias (7 items), sleep-disordered breathing (3 items), and daytime sleepiness (8 items).

Each item is rated on a 3-point Likert scale [(1=rarely (0-1 time/week), 2=sometimes (2-4 times/week), and 3=usually (5-7times/week)]. Subscale scores are calculated by summing the relevant items, with higher scores indicating greater sleep disturbance. The total CSHQ score ranges from 33 to 99, and a cutoff score of ≥41 was used to indicate clinically significant sleep disturbances, in accordance with the original validation study.

The CSHQ has demonstrated good reliability and validity in both typically developing children and children with neurodevelopmental disorders, including ASD. The full version was used in this study to allow a comprehensive assessment across multiple sleep domains, as recommended in prior ASD sleep research. A copy of the questionnaire used in this study is available from the corresponding author upon reasonable request.

Sample size calculation

The sample size was calculated using the standard formula for estimating a proportion in a cross-sectional study. In text form, the formula is: n equals Z squared multiplied by p multiplied by (1 minus p), divided by d squared. In this formula, n represents the required sample size, Z corresponds to a 95% confidence level (1.96), p represents an anticipated prevalence of sleep disturbances among children with autism spectrum disorder, conservatively set at 0.80 to reflect the high burden of sleep problems in this population, and d represents the desired margin of error (set at 0.07). Based on these parameters, the estimated sample size was approximately 126 participants.

During the study period, 132 eligible caregivers were approached, of whom 116 consented and completed the questionnaire. Although the achieved sample size was slightly smaller than the estimated target, 116 participants were considered adequate for the planned multivariable regression analyses based on commonly accepted subject-to-variable ratios.

Data analysis

All statistical analyses were performed using IBM SPSS Statistics for Windows, Version 26.0 (IBM Corp., Armonk, New York, USA). Continuous variables were summarized as mean±standard deviation, and categorical variables as frequencies and percentages. Normality was assessed using the Shapiro-Wilk test. Group comparisons were conducted using independent-samples t-tests or one-way analysis of variance for continuous variables and chi-square tests for categorical variables. Variables with a p-value<0.10 in bivariate analyses were entered into a multivariable linear regression model to identify independent predictors of sleep disturbance. Regression coefficients, 95% confidence intervals, and p-values were reported, with statistical significance set at p<0.05.

## Results

A total of 116 children with autism spectrum disorder were included in the analysis. The mean age of the children was 6.4±2.3 years. Nearly half of the participants were aged 3-6 years (n=52, 44.8%) or 7-10 years (n=53, 45.7%), while 11 children (9.5%) were older than 10 years. The sample was predominantly male (n=90, 77.6%). Autism severity, as reported by caregivers based on prior clinical assessment and physician communication, was categorized as moderate in 54 children (46.6%), mild in 31 children (26.7%), and severe in 11 children (9.5%). Twenty caregivers (17.2%) reported uncertainty regarding the severity level, reflecting variability in caregiver understanding of clinical severity classifications. Severity categories were based on prior clinical assessments conducted by treating physicians and communicated to caregivers during routine clinical follow-up, rather than on research-administered psychometric testing. Less than half of the children were receivingpharmacological treatment (n=49, 42.2%), most commonly risperidone (n=31, 63.3%), followed by aripiprazole (n=4, 8.2%) and atomoxetine (n=3, 6.1%). Eleven responses (22.4%) did not specify the medication name (Table 1).

**Table 1:** Bio-Demographic Characteristics of the study Children with Autism, Al-Ahsa, Saudi Arabia (N=116) This table presents the demographic and clinical background of the participating children, including age distribution, sex, nationality, age at autism diagnosis, autism severity, and treatment status. Medication types used by children receiving treatment are also shown. Values are expressed as frequencies and percentages (n, %).

Bio-Demographic data	Number	%
Child's age in years		
3-6	52	44.8%
7-10	53	45.7%
>10	11	9.5%
Child gender		
Male	90	77.6%
Female	26	22.4%
Age of diagnosis with autism		
Between 2-4 years	101	87.1%
Between 5-7 years	14	12.1%
Between 8-10 years	1	0.9%
The severity or degree of autism		
Mild	31	26.7%
Moderate	54	46.6%
Severe	11	9.5%
Not known	20	17.2%
Medication for autism		
Yes	49	42.2%
No	67	57.8%
If yes, name of the drug received		
Aripiprazole	4	8.2%
Atomoxetine	3	6.1%
Not reported	11	22.4%
Risperidone	31	63.3%

Family and lifestyle characteristics are summarized in Table 2. A family history of autism was reported in 34 children (29.3%). Parental consanguinity was present in 58 children (50.0%). Sleep-related difficulties were common, with 72 caregivers (62.1%) reporting anxiety or challenges related to the child’s sleep. Regarding screen exposure, 44 children (37.9%) used electronic devices for more than three hours daily, while 38 (32.8%) were limited to less than two hours. Most children (n=77, 66.4%) did not consume caffeine, although 30 children (25.8%) consumed caffeine either daily or every two to three days. Caffeine exposure refers to children's consumption of caffeine-containing products, such as chocolate, cola, tea, or energy-containing beverages, rather than adult caffeine intake. Frequency of consumption was recorded based on the caregiver's report of the child’s typical weekly intake. Physical activity levels were generally low, as 99 children (85.3%) exercised fewer than three days per week. In terms of sleep environment, 55 children (47.4%) slept with their parents, 43 (37.1%) slept with siblings, and only 18 (15.5%) slept alone. Additionally, 44 children (37.9%) used electronic devices before bedtime, and 27 (23.3%) had been given sleep medication at some point.

**Table 2:** Family, Lifestyle, and Sleep-Related Characteristics of Children with Autism in Al-Ahsa, Saudi Arabia (N=116) This table summarizes family-related factors (such as family history of autism and parental consanguinity), lifestyle behaviors (electronic device use, caffeine intake, physical activity), and sleep-related characteristics (parental sleep concerns, bedtime environment, screen use before sleep, sleeping arrangement, light exposure during sleep, and use of sleep medication). Data are presented as frequencies and percentages (n, %).

Factors	Number	%
Family history of autism		
Yes	34	29.3%
No	82	70.7%
Consanguinity of the parents		
Yes	58	50.0%
No	58	50.0%
Experience of anxiety or finding difficulty for the child to sleep		
Yes	72	62.1%
No	44	37.9%
The average daily usage of electronic devices or television viewing		
<2 hours	38	32.8%
2-3 hours	34	29.3%
>3 hours	44	37.9%
Daily consumption of caffeine-containing substances per week		
Not used at all	77	66.4%
<2 days	9	7.8%
every 2-3 days	15	12.9%
Daily used	15	12.9%
Playtime a week		
<3 days	99	85.3%
>3 days	17	14.7%
The child usually sleeps with		
With brothers	43	37.1%
With parents	55	47.4%
Alone	18	15.5%
Usage of electronic devices before bedtime		
Yes	44	37.9%
No	72	62.1%
Light of the room is on when the child goes to sleep		
Yes	27	23.3%
No	89	76.7%
Usage of any sleep-inducing medication before		
Yes	27	23.3%
No	89	76.7%

**Figure 1:**
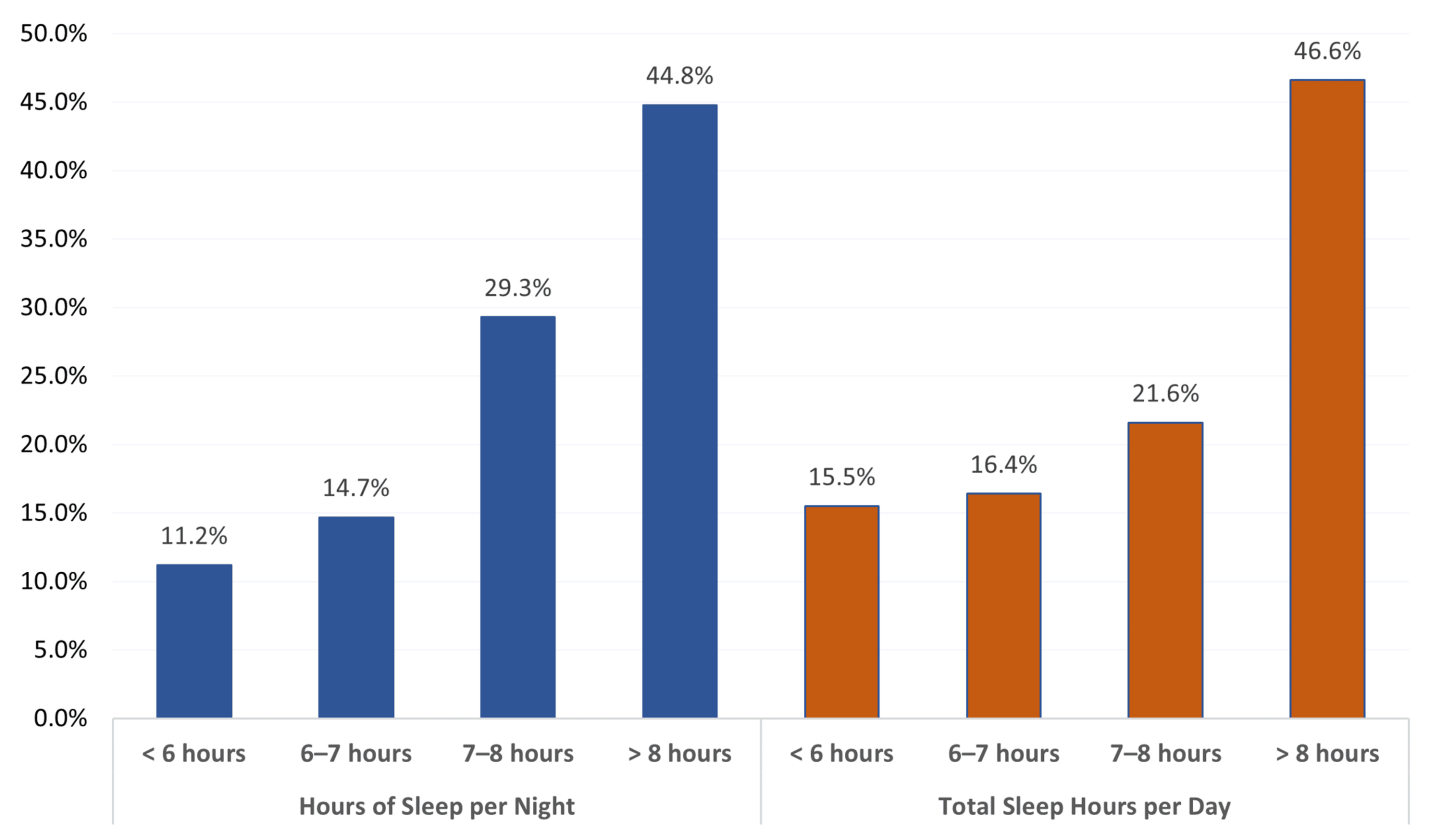
Sleep Hours (Day and Night) Among Children with Autism in Al-Ahsa, Saudi Arabia This figure illustrates the number of hours children slept during the night and their total daily sleep duration, including naps. Bars represent the proportion of children falling into different sleep-duration categories (<6 hours, 6–8 hours, and >8 hours), allowing visualization of sleep variability within the population.

Figure 1 illustrates the distribution of nighttime sleep hours and total daily sleep hours (including naps). During the night, 52 children (44.8%) slept more than eight hours, while 13 children (11.2%) slept less than six hours. When total daily sleep was assessed, the proportion of children sleeping more than eight hours increased to 54 (46.6%), while the percentage sleeping less than six hours rose to 18 (15.5%). These findings demonstrate considerable variation in sleep duration among children with autism.

Table 3 summarizes the domain scores of the CSHQ. Bedtime resistance showed a mean score of 11.2±2.2, reflecting frequent challenges at bedtime. Sleep-onset delay averaged 1.9±0.9, indicating difficulty initiating sleep among many children. The mean sleep duration domain score reflects caregiver-reported concerns related to insufficient sleep and does not represent actual sleep duration in hours; higher scores indicate greater perceived sleep insufficiency. Sleep anxiety was elevated (mean 8.6±1.6), and night waking was common (mean 7.6±1.5). Parasomnias had a mean score of 16.5±3.0, reflecting behaviors such as sleepwalking or night terrors. Sleep-disordered breathing had a mean score of 7.7±1.3, while daytime sleepiness was relatively high, with a mean of 19.0±3.0. Overall, total CSHQ scores ranged from 39 to 96, with a mean of 76.3±9.3, indicating a high burden of sleep disturbances in the study cohort.

**Table 3:** CSHQ Domain Scores (Range, Mean, and Standard Deviation) Among Children with Autism Spectrum Disorder in Al-Ahsa, Saudi Arabia (N=116) This table presents the domain scores of the Children’s Sleep Habits Questionnaire (CSHQ). All reported values represent summed subscale scores rather than objective sleep duration in hours. Higher scores across domains indicate greater severity of sleep-related problems, with elevated scores observed particularly in the bedtime resistance and daytime sleepiness domains.

Sleep domain	Min-Max	Mean±SD
Bedtime Resistance	6-17	11.2±2.2
Sleep Onset Delay	1-3	1.9±0.9
Sleep Duration	3-9	6.2±1.9
Sleep Anxiety	4-12	8.6±1.6
Night Waking	3-9	7.6±1.5
Parasomnias	7-21	16.5±3.0
Sleep Disordered Breathing	3-9	7.7±1.3
Daytime Sleepiness	9-24	19.0±3.0
Child sleep habit score	39-96	76.3±9.3

Table 4 presents unadjusted (bivariate) associations between child and family characteristics and total CSHQ scores. Significant differences in mean CSHQ scores were observed according to age at autism diagnosis, diagnosed autism severity, receipt of autism treatment, parental anxiety regarding the child’s sleep, caffeine consumption, sleeping arrangement, and history of sleep medication use. As higher CSHQ scores indicate greater sleep disturbance, lower mean scores reflect relatively fewer sleep problems in the corresponding groups. These findings represent unadjusted comparisons and do not account for potential confounding factors.

**Table 4:** Association of Child and Family Characteristics with Sleep Habit Scores Among Children with Autism Spectrum Disorder (Mean±SD) Data are presented as mean ± standard deviation. Analyses represent unadjusted (bivariate) comparisons. Independent-samples t-tests were used for dichotomous variables and one-way ANOVA for variables with more than two categories. Higher total CSHQ scores indicate greater sleep disturbance. P: one-way analysis of variance (ANOVA); #: independent samples t-test. * P<0.05 (significant).

Characteristic	Category	Mean ± SD	p-value
Child's age in years	3-6	76.9±10.4	.804
	7-10	76.0±8.6	
	>10	75.2±7.2	
Child gender	Male	76.5±9.8	.656^#^
	Female	75.6±7.7	
Age at autism diagnosis	Between 2-4 years	77.3±8.8	0.005*
	Between 5-7 years	70.9±10.2	
	Between 8-10 years	57.0±0.0	
Diagnosed autism severity	Mild	76.4±9.1	.049*
	Moderate	74.3±9.6	
	Severe	79.6±9.5	
	Not known	79.9±7.8	
Receiving autism treatments	Yes	73.5±10.3	.004*^#^
	No	78.4±8.0	
Family history of autism	Yes	74.6±10.3	.187^#^
	No	77.1±8.9	
Parental consanguinity	Yes	76.4±8.7	.945^#^
	No	76.3±9.9	
Parental anxiety about the child's sleep	Yes	74.8±10.4	.023*^#^
	No	78.8±6.7	
Daily screen time	<2 hours	75.0±11.7	.316
	2-3 hours	78.3±7.4	
	>3 hours	76.0±8.2	
Weekly caffeine consumption	Not used at all	77.1±9.4	.029*
	<2 days	80.3±6.9	
	Every 2-3 days	76.1±8.6	
	Daily	70.1±8.7	
Weekly exercise frequency	<3 days	76.5±9.2	.640^#^
	>3 days	75.4±10.0	
Child's usual sleeping arrangement	With brothers	78.6±9.5	.001*
	With parents	72.9±8.8	
	Alone	81.4±6.3	
Uses devices before bedtime	Yes	75.3±7.3	.360^#^
	No	77.0±10.4	
Sleeps with the light on	Yes	74.4±7.8	.212^#^
	No	76.9±9.7	
History of sleep medication use	Yes	71.1±11.6	.001*^#^
	No	77.9±7.9	

The multiple stepwise linear regression analysis identified several independent predictors of sleep habit scores, as shown in Table 5. Summarizes the results of the multiple stepwise linear regression analysis examining independent predictors of total CSHQ scores. After adjustment for covariates, age at autism diagnosis, receipt of autism treatment, parental anxiety regarding the child’s sleep, autism severity, sleeping alone, and history of sleep medication use remained significantly associated with total sleep habit scores. Differences in the magnitude and direction of associations compared with the bivariate analysis reflect adjustment for confounding variables and the coding of predictor reference categories in the regression model.

**Table 5:** Multiple Stepwise Linear Regression Analysis of Predictors of Sleep Habit Scores Among Children with Autism Spectrum Disorder Dependent variable: total CSHQ score. Higher scores indicate greater sleep disturbance. Regression coefficients represent adjusted associations relative to the stated reference category. P<0.05 indicates statistical significance. B: regression coefficient; SE: Standard error; * P<0.05 (significant).

Predictor	Reference category	B	SE	Beta	t	p-value
Age at autism diagnosis	Younger age	-7.01	2.25	-0.28	-3.11	.002*
Receiving autism treatment	No	2.57	1.02	0.14	1.49	.048*
Parental anxiety about sleep	No	3.98	1.59	0.21	2.50	.014*
Autism severity	Mild	1.19	0.50	0.14	-1.61	.049*
Child sleeps alone	No	1.87	0.98	0.14	-1.62	.048*
History of sleep medication use	No	6.47	2.08	0.29	3.11	.002*

## Discussion

In this sample of 116 children with ASD from Al-Ahsa, Saudi Arabia, we observed a substantial burden of sleep disturbances across multiple domains assessed using the CSHQ. Overall, our findings are consistent with existing literature and extend previous work by highlighting both universal sleep difficulties in ASD and region-specific patterns that may inform local clinical practice. Elevated bedtime resistance scores in our cohort suggest persistent behavioral challenges surrounding sleep initiation. This finding aligns with a multicenter Chinese survey in which bedtime resistance was the most frequently reported sleep disturbance among children with ASD [17]. Comparative studies between children with ASD and typically developing peers have similarly demonstrated significantly higher rates of bedtime resistance in ASD populations [18].

Sleep-onset delay was another prominent feature in our study. Sleep-onset delay refers to difficulty initiating sleep after bedtime and is commonly assessed through caregiver reports or objective sleep monitoring. Prior studies using actigraphy, a non-invasive objective method that measures sleep-wake patterns through a wearable motion-sensing device, have demonstrated that parent-reported sleep-onset delay correlates with prolonged sleep latency and increased night waking in children with ASD [19]. Multicenter data further report onset-delay problems in approximately 17.9% of children with ASD [17], supporting the relevance of this domain. Regarding sleep duration, it is important to clarify that the CSHQ sleep duration domain reflects caregiver-reported concerns related to insufficient sleep, rather than objective sleep duration measured in hours. Elevated sleep duration domain scores in our cohort, therefore, indicate perceived inadequacy of sleep rather than quantified sleep time. Similar findings have been reported in previous CSHQ-based studies, including work by Souders et al., which showed that children with ASD experience reduced or more variable sleep duration compared with typically developing peers [20].

Sleep anxiety was also notably elevated in our sample, reflecting emotional distress surrounding bedtime. This finding is consistent with prior multicenter research identifying sleep anxiety as one of the most problematic sleep domains in children with ASD [17]. Emotional dysregulation and anxiety have been repeatedly linked to sleep difficulties in ASD, reinforcing the interconnected nature of behavioral and sleep disturbances [21]. Night waking and parasomnias were common in our cohort, indicating disrupted nocturnal sleep. Parasomnias, including night terrors, sleepwalking, and confusional arousals, are well documented in ASD and may reflect underlying neurodevelopmental differences in arousal regulation and sensory processing [22-25]. Similarly, elevated sleep-disordered breathing scores suggest that breathing-related sleep disruptions may contribute to poor sleep quality, a finding supported by prior studies reporting higher rates of sleep-disordered breathing in children with ASD compared with typically developing children [17,26].

Daytime sleepiness was also prominent, underscoring the functional impact of poor nighttime sleep. Previous research has shown that daytime sleepiness in children with ASD is associated with increased behavioral challenges and impaired daily functioning [18,27]. Overall, the high total CSHQ scores observed in this study indicate a substantial burden of sleep disturbances. This finding is consistent with earlier studies reporting that more than two-thirds of children with ASD score above the clinical cutoff on the CSHQ [20,28-30]. Regional studies from Saudi Arabia similarly report shortened sleep duration, increased daytime sleepiness, and a high prevalence of sleep-related symptoms among children with ASD [31,32]. Sociocultural factors, family sleep practices, environmental conditions, and limited access to structured sleep interventions may contribute to the high prevalence observed in this region, highlighting an important area for future research.

In multivariable analysis, several factors remained independently associated with greater sleep disturbance. Later age at autism diagnosis was associated with worse sleep outcomes, suggesting a potential protective role of earlier diagnosis and intervention. Greater ASD severity was also independently associated with increased sleep disturbance, consistent with previous findings linking core symptom burden to sleep dysfunction. Importantly, parental anxiety regarding the child’s sleep emerged as an independent predictor, underscoring the bidirectional relationship between caregiver psychological well-being and child sleep. Addressing caregiver anxiety may therefore represent a modifiable target for improving sleep outcomes. Sleeping arrangements and prior use of sleep medications also remained significant predictors in the adjusted model. These findings should be interpreted as indicators of more complex or persistent sleep difficulties rather than causal factors. Specifically, a history of sleep medication use likely reflects more severe sleep pathology, emphasizing the need for careful monitoring and combined behavioral and environmental interventions rather than reliance on pharmacologic therapy alone. Clinically, these findings have several important implications. The high burden of sleep disturbances across multiple domains supports the routine incorporation of sleep assessment into standard ASD evaluations. Given the prominence of bedtime resistance, sleep anxiety, and sleep-onset delay, early behavioral sleep interventions-such as sleep hygiene education and parent coaching-should be integral components of ASD care. Addressing caregiver anxiety through education and psychosocial support may further improve sleep outcomes. Finally, early ASD diagnosis and continuous follow-up may confer benefits not only for developmental outcomes but also for secondary domains such as sleep.

This study has several strengths, including the use of a validated sleep assessment tool and a comprehensive evaluation of clinical, behavioral, and environmental factors in a regional ASD population. However, several limitations should be acknowledged. The cross-sectional design precludes causal inference. Data were based on caregiver-reported questionnaires, introducing potential recall and reporting bias. The study sample was drawn from a single center, which may limit generalizability to the broader ASD population in Saudi Arabia. Additionally, objective sleep measures such as actigraphy or polysomnography were not used, which could have provided a more precise characterization of sleep patterns.

Despite these limitations, the findings provide valuable insights and highlight the need for future longitudinal and multi-center studies incorporating objective sleep measures to better understand sleep trajectories and intervention outcomes in children with ASD.

## Conclusions

This study demonstrates a high burden of sleep disturbances among children with autism spectrum disorder in Al-Ahsa, Saudi Arabia, affecting multiple sleep domains, including bedtime resistance, sleep-onset delay, sleep anxiety, night waking, parasomnias, and daytime sleepiness. While direct comparisons with typically developing children were not performed, the magnitude and breadth of sleep difficulties observed are comparable to, and in some domains exceed, those reported in previous international and regional studies of children with ASD.

Multivariable analysis identified later age at autism diagnosis, greater ASD severity, parental anxiety regarding the child’s sleep, sleeping arrangement, and prior use of sleep-inducing medications as independent factors associated with greater sleep disturbance. These findings highlight the multifactorial nature of sleep problems in ASD and underscore the importance of early diagnosis, routine sleep screening, caregiver support, and integrated behavioral and medical approaches within autism care programs.
